# Trimodal Therapy for Primary Squamous Cell Carcinoma of the Vagina: A Case Report

**DOI:** 10.7759/cureus.116053

**Published:** 2026-09-10

**Authors:** Jake Singh, Thomas R Eanelli

**Affiliations:** 1 Radiation Oncology, Touro College of Osteopathic Medicine, Middletown, USA; 2 Radiation Oncology, Garnet Health, Middletown, USA

**Keywords:** brachytherapy, external beam radiation therapy, gynecologic malignancy, high-dose-rate (hdr) brachytherapy, vaginal squamous cell carcinoma

## Abstract

Primary vaginal squamous cell carcinoma (SCC) is a rare clinical entity, with few prospective studies regarding its treatment available in the literature. We report a case of a 64-year-old female patient with a two-year history of vulvar itching, burning, and intermittent vulvar bleeding. She was diagnosed with stage III (T2, N1, M0) vaginal SCC. The patient subsequently received chemotherapy with cisplatin, external beam radiation therapy (EBRT), as well as high-dose-rate (HDR) cylinder brachytherapy. She achieved a complete clinical and radiographic response, with no evidence of disease on examination or post-treatment PET at six-month follow-up. This case highlights a multimodal treatment approach for advanced vaginal SCC and contributes to the limited body of literature guiding management of this rare malignancy.

## Introduction

Primary vaginal cancer represents between 1% and 2% of all gynecological malignancies [1,2]. The incidence rate of vaginal cancer in the United States is approximately 0.7 per 100,000 woman-years, with a median age at diagnosis of 69 years for invasive disease [3]. Notably, the incidence of invasive vaginal squamous cell carcinoma (SCC) increases continuously with advancing age, with peak rates observed in the 85- to 89-year age cohort, while in situ SCC peaks at approximately age 70 years and then declines [2]. Globally, vaginal cancer incidence rates are low and relatively stable. An analysis of population-based cancer registry data from 68 countries (1983-2012) found only a threefold variation in vaginal cancer incidence rates across countries, in contrast to the 30-fold variation observed for vulvar cancer [4].

The rare nature of vaginal cancer makes phase III trials prohibitive, limiting the majority of information available to retrospective studies and studies done on cervical or vulvar cancer. The anatomical considerations of vaginal cancer, including proximity to the rectum, bladder, and female urethra, can profoundly impact the patient’s physical and mental health. Severe late toxicity, predominantly gastrointestinal and genitourinary complications, has been reported in 8-30% of patients treated with definitive radiotherapy, and vaginal morbidity, including stenosis and mucosal ulceration, is likely underestimated [5]. These considerations underscore the importance of individualized, multidisciplinary treatment planning and the need for continued contributions to the literature to guide the management of this rare malignancy.

## Case presentation

We present a 64-year-old female who presented in November 2024 with a two-year history of vulvar burning and an itching sensation with intermittent vulvar bleeding. The patient has a remote history of cervical dysplasia treated with cryotherapy procedures in 1988 and 1994. She has no family history of gynecological cancer. Her relevant past surgical history is significant for CO_2_ laser ablation, hysterectomy, and vulvectomy for vulvar intraepithelial neoplasia (VIN) 2-3.

In January 2025, physical examination revealed a firm, irregular, exophytic mass involving the left vaginal sidewall and distal left vagina, with an ulcerated, friable surface that bled readily on contact. The lesion had rolled, indurated margins. Examination of the vulva showed post-surgical changes consistent with prior vulvectomy, without evidence of a grossly visible recurrent vulvar lesion. Bimanual examination did not reveal gross parametrial or pelvic sidewall fixation, and no inguinal lymphadenopathy was appreciated on palpation. Biopsy of the left vaginal sidewall and distal left vagina revealed squamous cell carcinoma. The specimen demonstrated superficially invasive squamous cell carcinoma, not otherwise specified. Immunohistochemistry revealed strong and diffuse staining for p16, suggestive of a human papillomavirus (HPV)-associated cancer.

In March 2025, a PET/CT scan revealed a large, multilobulated, PET-avid 4.8x2.6x3.9 cm vaginal mass (Figure 1). The mass extended to the base of the bladder, partially surrounding the female urethra, with further extension to the anterior wall of the rectum and inferior aspect of the left pubic symphysis. A 10 mm PET-avid lymph node was detected near the right paravaginal/vaginal cuff. Based on these findings, she was diagnosed with International Federation of Gynecology and Obstetrics (FIGO) stage III (T2, N1, M0) vaginal SCC.

**Figure 1:**
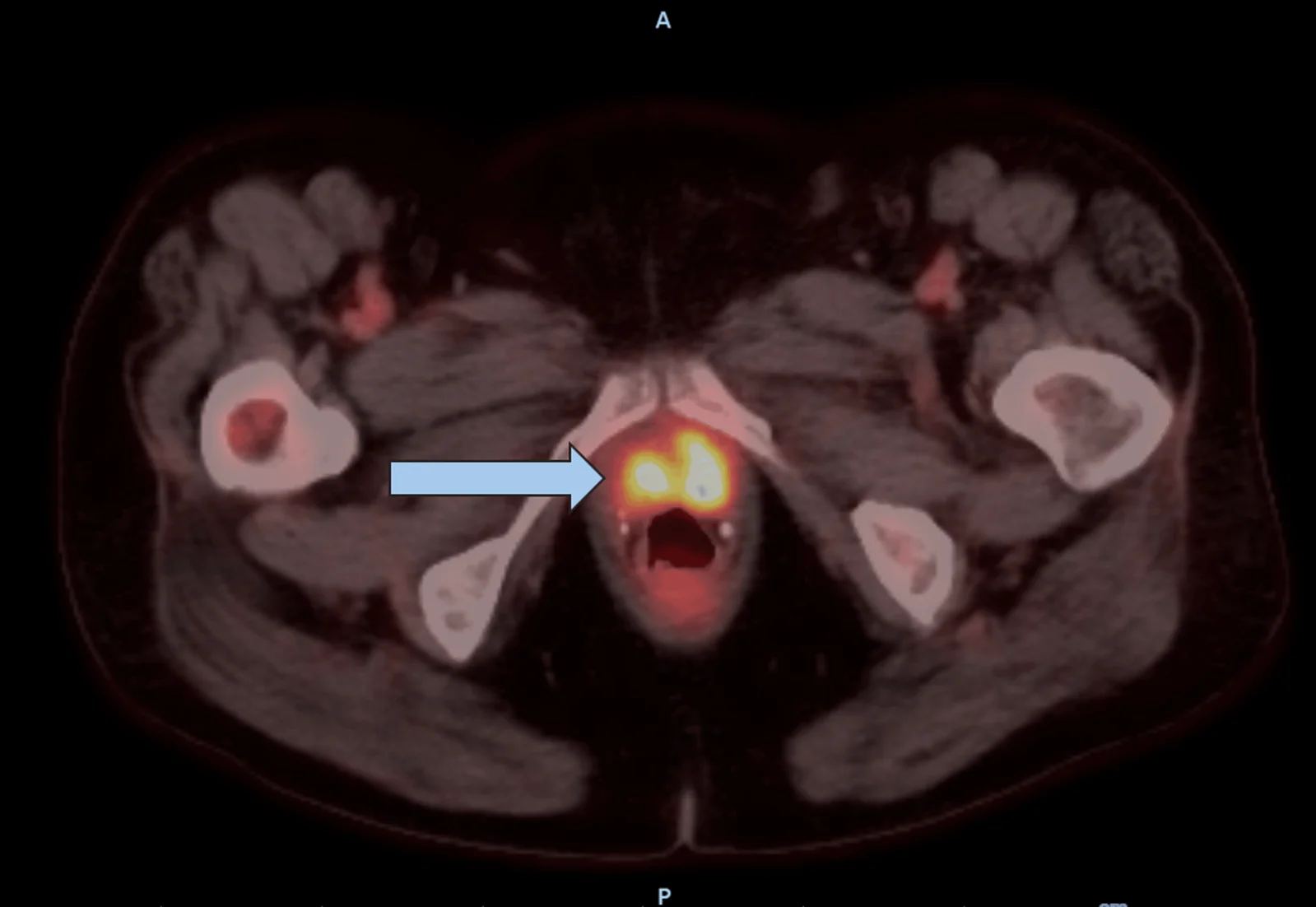
Pre-treatment PET scan identifying gross tumor volume (arrow).

Given her prior history of cervical dysplasia and VIN 2-3, a concurrent cervical or vulvar primary malignancy was specifically considered and excluded. The patient had undergone prior hysterectomy and therefore had no cervix, precluding a cervical primary. Examination of the vulva demonstrated only post-surgical changes from her prior vulvectomy, with no grossly visible invasive vulvar lesion, and the tumor did not extend across the introitus to involve the vulva. On PET/CT, the mass was centered within the vagina, involving the left vaginal sidewall and distal vagina. The lesion was therefore classified as a primary vaginal carcinoma.

Therapeutic intervention

The patient received cisplatin chemotherapy, external beam radiation therapy (EBRT), as well as high-dose-rate (HDR) cylinder brachytherapy. During her five weeks of radiation therapy, she received five cycles of 40 mg/m^2^ cisplatin chemotherapy, dosed one week apart.

Treatment plan for EBRT

Her EBRT was given daily, five days a week. The treatment was given on a Varian 21iX linear accelerator (Palo Alto, CA: Varian Medical Systems). She received 25 fractions utilizing volumetric modulated arc therapy/intensity-modulated radiation therapy/image-guided radiation therapy/simultaneous integrated boost (VMAT/IMRT/IGRT/SIB) planning. The gross tumor volume, encompassing the primary vaginal tumor and the suspicious 10 mm right paravaginal/vaginal cuff nodule, received a total of 50 Gy. The elective clinical target volume, encompassing the perineum, paravaginal region, and pelvic lymph nodes, received 45 Gy. The suspicious nodule therefore received an additional 5 Gy relative to the surrounding elective nodal volume via the simultaneous integrated boost. A total of 6 MV photons were prescribed. Imaging for the EBRT treatment plan is shown in Figure 2.

**Figure 2:**
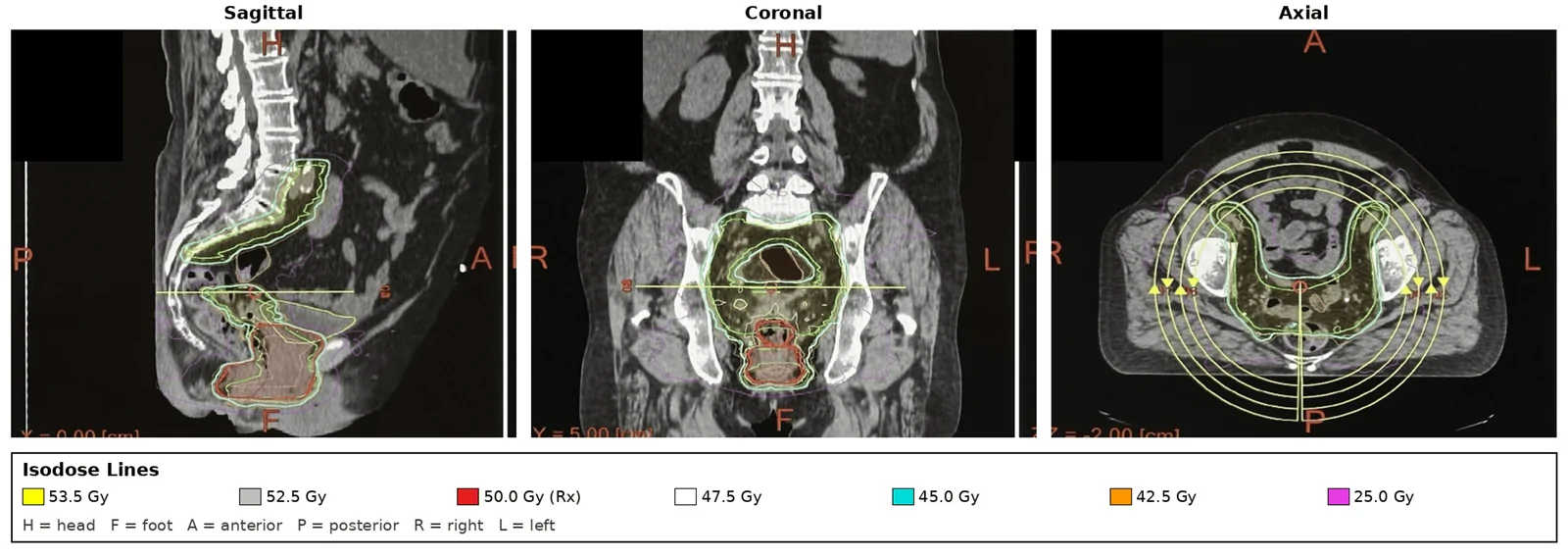
External beam dosimetry plan. Sagittal, coronal, and axial CT images through the pelvis showing the isodose distribution for the external beam radiation therapy (EBRT) plan, delivered using VMAT/IMRT/IGRT with a simultaneous integrated boost (SIB) technique. Isodose lines are labeled in Gy; the 50 Gy line corresponds to the prescription dose. The green/cyan contour outlines the planning target volume (PTV) encompassing the vaginal tumor and clinical nodal target volume. The bladder and rectum are visualized as organs at risk (bladder, coronal panel; rectum, sagittal and coronal panels, outlined in orange/red). The concentric yellow circles on the axial image represent the VMAT arc gantry trajectory used to deliver the plan. VMAT: volumetric modulated arc therapy; IMRT: intensity-modulated radiation therapy; IGRT: image-guided radiation therapy

Treatment plan for cylinder

The patient was examined weekly during EBRT, with progressive tumor regression documented at each on-treatment visit. By completion of EBRT, the tumor had achieved a complete clinical response, subsequently confirmed on post-treatment MRI. Given this complete response, there was initial uncertainty as to whether interstitial or intracavitary HDR brachytherapy would be optimal, and the patient was referred to a tertiary care center with an established brachytherapy service for further evaluation. Following a joint review between the treating institutions and a discussion at a multidisciplinary gynecologic oncology tumor board, consensus was reached that intracavitary cylinder brachytherapy was sufficient, as interstitial brachytherapy is reserved for residual disease that is too thick, bulky, or asymmetric for an intracavitary applicator to cover adequately, and no measurable residual disease remained.

Her cylinder brachytherapy was given weekly. In each instance, we used a 2.5 cm cylinder prescribed to the cylinder surface, and the patient was given 7 Gy with the prescription point at the cylinder surface, except at the tip, where the prescription point was 0.5 cm. The active length was 7 cm. The imaging for the cylinder treatment plan is shown in Figure 3.

**Figure 3:**
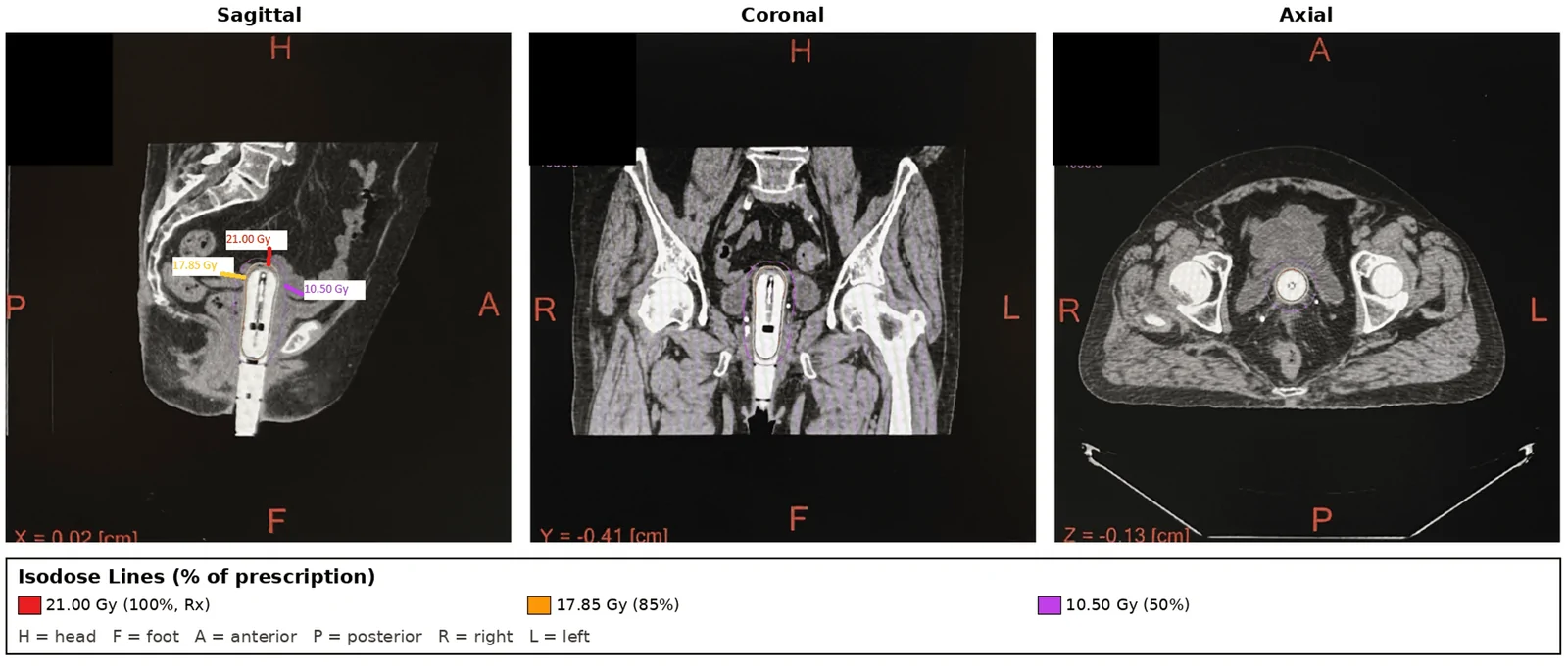
Dosimetric treatment plan for cylinder brachytherapy. Sagittal, coronal, and axial CT images through the pelvis showing the isodose distribution for the high-dose-rate (HDR) vaginal cylinder brachytherapy plan. A 2.5 cm diameter cylinder applicator was used, with an active length of 7 cm. Isodose lines are expressed in Gy as a percentage of the prescription dose, which was 7 Gy per fraction to the cylinder surface (0.5 cm depth at the tip), delivered weekly over three fractions for a cumulative prescribed dose of 21 Gy (100% line, red). The axial image demonstrates the circumferential dose fall-off from the cylinder surface.

Cumulative equivalent dose in 2 Gy fractions (EQD2) to the tumor, as well as detailed brachytherapy dose-volume parameters for target coverage and organ-at-risk (bladder, rectum, urethra) doses, were not calculated as part of routine clinical treatment planning and are therefore not available for reporting.

Follow-up and outcomes

The patient was seen at three-month and six-month post-treatment intervals. Physical examination revealed complete resolution of the previously noted vaginal mass, with no palpable or visually apparent residual lesion at the left vaginal sidewall or distal vagina. The vaginal mucosa demonstrated expected post-radiation changes, including pallor, thinning, and scattered telangiectasias, with loss of normal rugation. Mild vaginal shortening and narrowing consistent with post-brachytherapy fibrosis were noted, without evidence of complete stenosis or synechiae formation. The vulva showed stable post-surgical changes from prior vulvectomy without new lesion. Bimanual and rectovaginal examination revealed no palpable mass, induration, or nodularity along the rectovaginal septum, and no fixation to the pelvic sidewall. No inguinal or pelvic lymphadenopathy was appreciated on palpation. Figure 4 shows a post-treatment PET scan demonstrating complete radiographic resolution of the disease.

**Figure 4:**
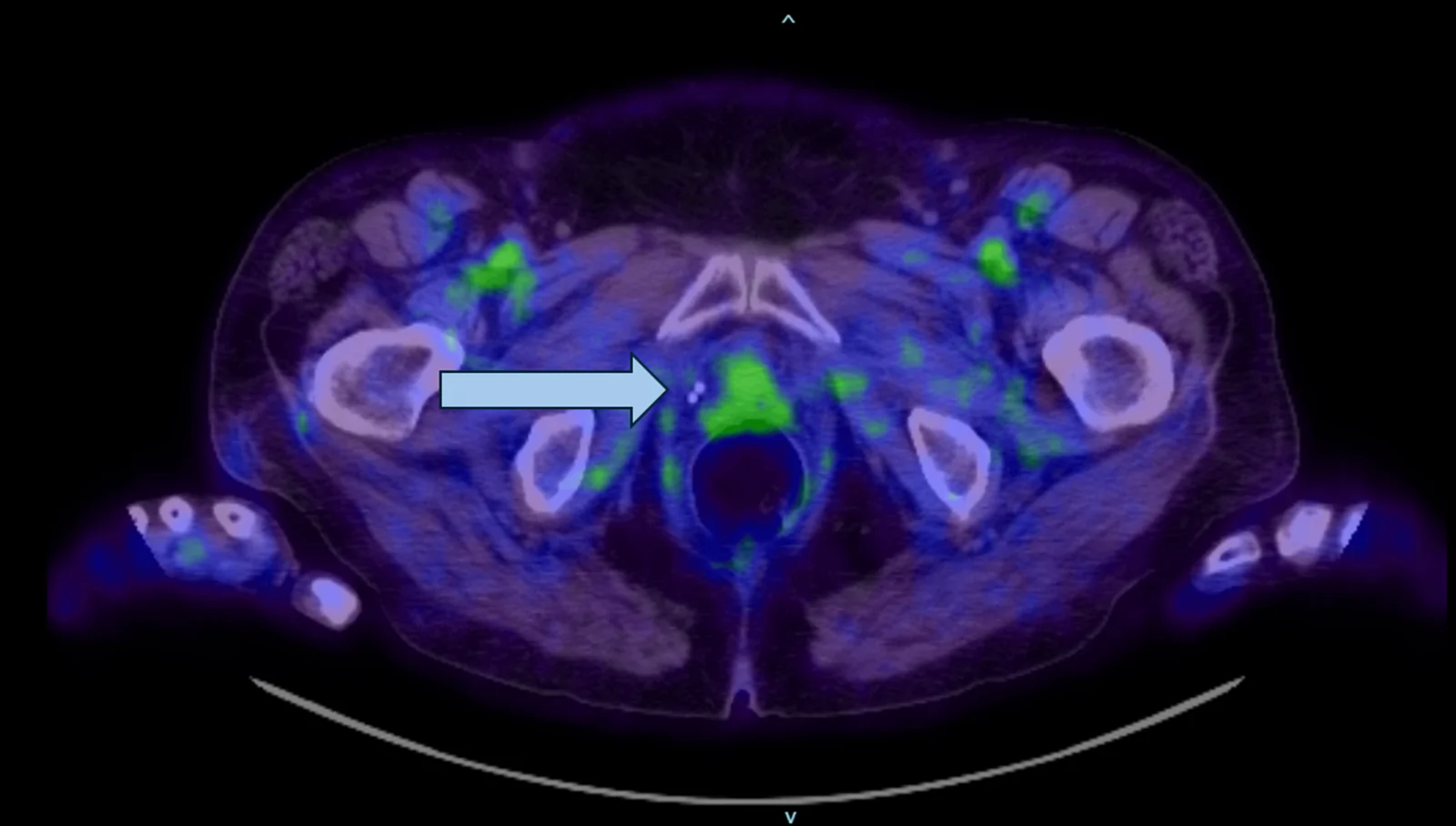
Post-treatment PET scan demonstrating complete response to chemoradiation with post-treatment changes. The arrow indicates the site of the previously identified gross tumor volume, with complete radiographic resolution of the disease.

## Discussion

Primary vaginal cancer is rare, accounting for only 1-2% of all gynecologic cancers [1,2]. Table 1 summarizes incidence rates for all gynecologic malignancies [3]. Of the primary vaginal cancers, squamous cell carcinoma is the most common histology, representing between 80% and 90% of these malignancies. Adenocarcinoma represents between 5% and 10% of malignancies, with other histologies such as neuroendocrine carcinoma and melanoma making up the remainder of diagnoses [6].

**Table 1:** Incidence rates of gynecological malignancies in the United States, 2018-2022.

Cancer site	Rate per 100,000 woman-years	Percentage of gynecological malignancies
Cervix uteri	7.7	15.5
Corpus and uterus	28.3	57.1
Ovary	10.3	20.8
Vagina	0.7	1.4
Vulva	2.6	5.2

Risk factors for vaginal SCC include high-risk human papillomavirus (HPV) infection, with HPV16 being the most predominant form [7,8]. One retrospective study of 593 patients with vaginal SCC showed that 66.7% of patients had a high-risk strain of HPV [9]. It is more common in older, post-menopausal women, reflecting the accumulation of risk factors over time. Like many malignancies, tobacco smoking and immunosuppression are strongly associated with the risk of developing vaginal cancer [10].

The most common symptom of vaginal cancer is painless vaginal bleeding and discharge [10]. In more advanced disease, it can impact nearby organs, leading to urinary tract symptoms such as dysuria, urinary retention, and hematuria [10]. Tumors invading the posterior vaginal wall may produce gastrointestinal symptoms such as constipation or hematochezia [10].

The preferred imaging modality for pretreatment assessment and recurrent vaginal cancer is MRI. PET/CT is used for detecting nodal and distant metastases, as well as recurrent disease [6]. While imaging findings are widely used to assess tumor size and guide therapy, the International Federation of Gynecology and Obstetrics (FIGO) guidelines note that they should not be used to change the initial clinical stage [1].

Staging is generally performed by combining the following two systems: the FIGO guidelines and the American Joint Committee on Cancer (AJCC) system [6]. According to FIGO criteria, a tumor involving the cervix must be classified as a cervical primary and one involving the vulva as a vulvar primary; primary vaginal carcinoma is in this respect a diagnosis of exclusion [1]. This distinction is particularly relevant in patients such as ours, with a prior history of HPV-related cervical and vulvar intraepithelial neoplasia, in whom multifocal or metachronous lower genital tract disease must be considered. Notably, p16 positivity and HPV association are shared across HPV-driven neoplasia of the cervix, vagina, and vulva, and therefore cannot by themselves localize the site of origin; the distinction rests on anatomic and clinical distribution. In this case, prior hysterectomy precluded a cervical primary, no invasive vulvar lesion was identified on examination, and the tumor was centered within the vagina without vulvar involvement. Our patient was therefore classified as having stage III (T2, N1, M0) primary vaginal SCC, with invasion of nearby structures and nodal metastasis near the vaginal cuff.

Tumor stage at the time of diagnosis is the strongest prognostic factor for vaginal SCC [11,12]. In one study of 124 patients, the five-year disease-specific survival (DSS) rates for vaginal cancer (including SCC and adenocarcinoma) were 84.3%, 73.7%, 78.6%, and 26.5% for stage I, II, III, and IV, respectively [13]. In another study of 91 patients with primary vaginal SCC, the two-year overall survival rates were 96.2%, 92.3%, 66.6%, and 25.0% for stages I, II, III, and IV, respectively [14]. Measures of tumor spread, including advanced stage (III/IV) disease, lymph node metastasis, and tumors larger than 4 cm, are all negative prognostic factors [15]. Tumors located outside the upper one-third of the vagina also carry poor prognostic value, potentially owing to greater proximity to organs such as the bladder and rectum [15]. Favorable prognostic factors for vaginal SCC include presence of high-risk HPV DNA, p16 positivity (used as a surrogate marker for HPV infection), and low Molecular Immunology Borstel-1 (MIB-1) index [7,15].

Tumor grade and degree of differentiation have also been studied as potential prognostic indicators. In a Surveillance, Epidemiology, and End Results (SEER)-based analysis of primary vaginal cancer, five-year cause-specific survival differed significantly by grade on univariate analysis (61.7%, 61.0%, 55.1%, and 36.2% for grade I through IV, respectively; grade IV vs. grade I, HR: 1.82, p=0.013), but grade did not retain independent significance once tumor, node, metastasis (TNM) stage, tumor size, and treatment modality were included in multivariate modeling [16]. This suggests that grade may correlate with disease behavior but is not established as an independent predictor of outcome apart from stage, underscoring that the absence of grading data in the present case, while a limitation, is less likely to materially affect interpretation of her outcome than would, for example, missing stage or HPV status.

According to the National Comprehensive Cancer Network (NCCN) Clinical Practice Guidelines, the treatment options for stage I vaginal SCC include radiation therapy or surgical resection [17]. Radiation therapy is preferred as a primary treatment due to the decreased risk of incidental organ damage and consists of intracavitary brachytherapy (preferred for cases with lesions <2 cm and thickness <5 mm) or EBRT with intracavitary brachytherapy [17]. Because of anatomical considerations, such as proximity to the bladder, surgical resection is considered only if complete resection with clear margins is possible and the likelihood of needing future adjuvant radiotherapy is low [14,17].

The NCCN guidelines recommend platinum-based chemoradiation with brachytherapy or EBRT with brachytherapy as the first-line treatment for stage II-IVA cancers [17]. Within this treatment paradigm, brachytherapy serves a distinct role as a boost to the primary tumor following EBRT, rather than as a stand-alone modality, in locally advanced disease. Analogous to its function in cervical cancer, a brachytherapy boost permits dose escalation to the vaginal tumor bed beyond what can be safely delivered by EBRT alone, while limiting dose to the adjacent bladder and rectum through the rapid dose fall-off characteristic of intracavitary and interstitial techniques [5]. Image-guided adaptive brachytherapy, using target concepts developed specifically for primary vaginal cancer, has been associated with improved local control when delivered without unnecessary treatment delay [5].

In 2024, a prospective study on vaginal cancer was performed on 21 patients with varying stages of disease using a standardized trimodal approach. Combining EBRT, cisplatin-based chemotherapy, and brachytherapy, the two-year actuarial local control, metastasis-free survival, and overall survival rates were 79.4%, 90.5%, and 79.4%, respectively [18].

Limitations

The most fundamental limitation is that this is a single case report. Findings from a single patient cannot be generalized to the broader population of patients with vaginal SCC, and no causal inferences can be drawn regarding treatment efficacy. The favorable outcome observed may reflect patient-specific factors, most notably her HPV-positive/p16-positive status, a known favorable prognostic marker, rather than the treatment approach itself. Without a comparator arm or a larger cohort, it is impossible to disentangle the relative contribution of tumor biology, patient factors, and treatment technique to the observed response [7,15].

Further pathologic characterization, including tumor grade and degree of differentiation, was sought but could not be obtained. The limited specimen supported a cytologic diagnosis but was insufficient for full histopathologic grading. Repeat biopsy was recommended to obtain adequate tissue; however, the patient, who had a severe alcohol-use disorder characterized by daily consumption, episodes of blackout, and multiple traumatic falls, declined to pursue further diagnostic evaluation. Consequently, tumor grade and degree of differentiation could not be determined, and the pathologic data available for this case remain limited to histologic subtype.

The follow-up period extends only to six months post-treatment. Given that vaginal SCC recurrences can occur years after initial treatment, the current follow-up is insufficient to draw conclusions about durable local control, disease-free survival, or overall survival. Longer-term surveillance, including the planned three- and six-month visits extending to annual follow-up, will be necessary to meaningfully assess durability of response and to capture late toxicity.

Related to this, the assessment of late treatment-related morbidity is similarly limited by the short observation window. Radiation-associated toxicities, including vaginal stenosis, mucosal ulceration, and genitourinary or gastrointestinal complications, are known to manifest or worsen over months to years following completion of therapy [5]. As such, this report cannot speak to the patient's long-term functional or quality-of-life outcomes, both of which are critical considerations in a malignancy located so close to the bladder, urethra, and rectum.

Additionally, because vaginal SCC is rare and disease-specific phase III data are lacking, this patient's treatment plan was extrapolated largely from cervical cancer protocols. While this is standard practice in the field given the paucity of vaginal-cancer-specific evidence, it introduces uncertainty as to whether the dosing, fractionation, and chemotherapy regimen used represent the optimal approach for vaginal primary disease specifically, as opposed to an adapted cervical cancer protocol.

Finally, as a single-institution case managed by a single multidisciplinary team, the treatment planning, imaging interpretation, and follow-up protocols reflect institution-specific practices and resources that may not be uniformly available or reproducible elsewhere, thereby limiting the external applicability of this approach.

## Conclusions

We presented a case of stage III (T2, N1, M0) primary vaginal SCC in a patient with a prior history of HPV-related lower genital tract neoplasia, treated with a trimodal approach of concurrent cisplatin-based chemotherapy, EBRT with volumetric modulated arc therapy/intensity-modulated radiation therapy/image-guided radiation therapy/simultaneous integrated boost (VMAT/IMRT/IGRT/SIB) planning, and HDR cylinder brachytherapy. She achieved a clinically and radiographically disease-free status at six-month follow-up, an outcome that may reflect favorable biologic features, HPV positivity, and p16 overexpression, as much as the treatment delivered.

Given the limited follow-up, this case should be viewed as a single illustrative data point rather than a demonstration of treatment efficacy. Continued longitudinal follow-up of this patient, alongside additional cases and pooled retrospective analyses, will be necessary to characterize durable outcomes, late toxicity, and optimal treatment sequencing, and to move the field beyond reliance on cervical cancer-derived protocols toward vaginal-cancer-specific standards of care.

## References

[1] Adams TS, Rogers LJ, Cuello MA (2021). Cancer of the vagina: 2021 update. Int J Gynaecol Obstet.

[2] Wu X, Matanoski G, Chen VW, Saraiya M, Coughlin SS, King JB, Tao XG (2008). Descriptive epidemiology of vaginal cancer incidence and survival by race, ethnicity, and age in the United States. Cancer.

[3] (2025). Recent trends in SEER age-adjusted incidence rates, 2000-2023. https://seer.cancer.gov/statistics-network/explorer/..

[4] Bray F, Laversanne M, Weiderpass E, Arbyn M (2020). Geographic and temporal variations in the incidence of vulvar and vaginal cancers. Int J Cancer.

[5] Westerveld H, Nesvacil N, Fokdal L (2020). Definitive radiotherapy with image-guided adaptive brachytherapy for primary vaginal cancer. Lancet Oncol.

[6] Kilcoyne A, Gottumukkala RV, Kang SK (2021). ACR appropriateness criteria staging and follow-up of primary vaginal cancer. J Am Coll Radiol.

[7] Tung H, Wang Y, Lin C (2024). Human papillomavirus prevalence, genotype distribution, and prognostic factors of vaginal cancer. Int J Cancer.

[8] Baral SK, Biswas P, Kaium MA (2022). A comprehensive discussion in vaginal cancer based on mechanisms, treatments, risk factors and prevention. Front Oncol.

[9] Bertoli HK, Rasmussen CL, Sand FL, Albieri V, Norrild B, Verdoodt F, Kjaer SK (2019). Human papillomavirus and p16 in squamous cell carcinoma and intraepithelial neoplasia of the vagina. Int J Cancer.

[10] Di Donato V, Bellati F, Fischetti M, Plotti F, Perniola G, Panici PB (2012). Vaginal cancer. Crit Rev Oncol Hematol.

[11] Jhingran A (2022). Updates in the treatment of vaginal cancer. Int J Gynecol Cancer.

[12] Daling JR, Madeleine MM, Schwartz SM (2002). A population-based study of squamous cell vaginal cancer: HPV and cofactors. Gynecol Oncol.

[13] Yang J, Delara R, Magrina J (2020). Management and outcomes of primary vaginal cancer. Gynecol Oncol.

[14] Hiniker SM, Roux A, Murphy JD, Harris JP, Tran PT, Kapp DS, Kidd EA (2013). Primary squamous cell carcinoma of the vagina: prognostic factors, treatment patterns, and outcomes. Gynecol Oncol.

[15] Gadducci A, Fabrini MG, Lanfredini N, Sergiampietri C (2015). Squamous cell carcinoma of the vagina: natural history, treatment modalities and prognostic factors. Crit Rev Oncol Hematol.

[16] Huang J, Cai M, Zhu Z (2020). Survival and prognostic factors in primary vaginal cancer: an analysis of 2004-2014 SEER data. Transl Cancer Res.

[17] Abu-Rustum NR, Campos SM, Amarnath S (2026). Vaginal cancer, version 2.2026, NCCN Clinical Practice Guidelines in Oncology. J Natl Compr Canc Netw.

[18] Lancellotta V, Macchia G, Garganese G (2024). Trimodal definitive invasive vaginal carcinoma treatment (TRIDENT protocol): how a standardized approach may change prognostic outcomes. Int J Gynecol Cancer.

